# Implementing Canada’s first national virtual phone based overdose prevention service: lessons learned from creating the National Overdose Response Service (NORS)

**DOI:** 10.1186/s12954-024-01017-7

**Published:** 2024-05-28

**Authors:** William Rioux, Pamela Taplay, Lisa Morris-Miller, S. Monty Ghosh

**Affiliations:** 1 Department of Medicine, Faculty of Medicine & Dentistry, University of Alberta, Edmonton, AB Canada; 2 Grenfell Ministries, Hamilton, ON Canada; 3 Department of Internal Medicine, Faculty of Medicine & Dentistry, University of Alberta, Edmonton, AB Canada

## Abstract

The opioid epidemic remains one of the largest public health crises in North America to date. While there have been many diverse strategies developed to reduce the harms associated with substance use, these are primarily concentrated within a few large urban centers. As a result, there have been increased calls for equitable access to harm reduction services for those who cannot or choose not to access in-person harm reduction services. In December 2020, Canada’s National Overdose Response Service (NORS) a telephone based overdose response hotline and virtual supervised consumption service, was established in collaboration with various agencies and people with lived and living experience of substance use (PWLLE) across Canada to expand access to harm reduction services using novel Opioid Response Technology. In this manuscript we explore the lessons learned from the establishment and continued operation of the service exploring topics related to the initial establishment of the service, securing a phone line, routing technology, EMS dispatch solutions, peer and volunteer recruitment, legal and ethical support, policy and procedure development, securing funding, and marketing. Furthermore, we detail how this service has grown and changed in response to the various needs of service users.

## Background

The opioid crisis remains one of the largest public health challenges facing Canada and broader North America after the declared end to the COVID-19 pandemic [1, 2]. In efforts to address the continued rise in morbidity and mortality from this epidemic, supervised consumption sites, supervised injection facilities, and overdose prevention services have been implemented in various urban centers in Canada to reduce the harms associated with illicit drug use [3, 4]. Services designed to prevent harm among people who use drugs are essential to reduce the transmission of infectious diseases, reduce public drug use, and most importantly, reduce overdose deaths while remaining cost-effective [5]. Indeed, using drugs by oneself has been linked to significantly higher rates of fatal overdose [6]. Despite the benefits of supervised consumption sites, there continues to be a lack of these services in large portions of Canada and the United States due to issues such as political will, funding, and public understanding of the importance of these services [7].

The COVID-19 pandemic has only served to exacerbate the opioid epidemic with mandatory isolation guidelines doubling opioid-related mortality rates in Canada [1, 8]. Due to these various restrictions, many vital harm reduction services had to close their doors or significantly limit their hours, resulting in accessibility issues [9]. This pandemic also demonstrated continued access challenges for individuals outside of large urban centers and mid-sized cities [10]. Geospatial analyses of overdose deaths in the province of British Columbia, Canada, highlight a 30% increase in rate of fatal overdose in rural settings, partially attributed to the lack of harm reduction services and the unpredictable drug supply challenges in these areas [11].

Due to surging numbers of overdoses resulting from an increasingly toxic drug supply compounded by public health messaging promoting isolation, which ran directly counter to previous messaging to “never use alone”, novel strategies were explored including the use of overdose response technologies [12–17]. One innovative strategy, “spotting” is a method in which individuals are able to supervise clients while using substances virtually through the use of telecommunications and enact a response (which includes notifying a trusted community member or emergency medical services to intervene and provide naloxone) in the event of an overdose [18]. Various more formalized services have thus emerged from this grassroots movement, including cell phone applications and overdose response hotlines (OPH) [19]. These provide similar spotting services and act as a virtual counterpart to physical supervised consumption services extending the reach and accessibility of harm reduction across Canada and the United States. As a whole, these fall under the broader category of Mobile Overdose Response Services (MORS).  These services have demonstrated early effectiveness in reducing morbidity and mortality related to substance use [18–22], as well as some cost benefit [23]. Based on initial qualitative evaluations, there has been reasonable support for these services from health care providers and individuals using substances, with no particular preference for the type of service used between phone line and app-based services [19, 24–27]. This manuscript aims to describe the lessons learned from the establishment of Canada’s first-ever national ORH, the National Overdose Response Service (NORS) [28]. This service aimed to reduce the morbidity and mortality resulting from illicit substance use and expand access to harm reduction services beyond urban centers in which brick-and-mortar harm reduction services are traditionally concentrated [7]. We describe the strengths and limitations of this service and outline additional measures that should be undertaken to ensure continued efficacy and success.

## Methods

The following section is divided into an overview of the service, followed by pre-launch implementation concerns, overview of implementation processes, and a post-launch implementation discussion.

### Overview of the service

NORS was established in December 2020 as a collaborative of existing overdose response hotlines and apps in Canada, namely the Overdose Prevention Line from Grenfell Ministries, Brave Co-Op in Vancouver British Columbia [29] and Alberta Health Services in Calgary [30]. Due to the impact of the dual public health crises from the pandemic and opioid crisis, the line was launched before securing funding. Clients who call into the service are connected to an operator who will provide a brief introduction to the service, gather basic information including a unique caller code, created by the client through a combination of first and last name and year of birth, -their substances used, amounts, and routes of administration, and optionally their gender and indigeneity. Furthermore, clients and operators co-create an emergency response plan should the individual become unresponsive. This consists of collecting the individual address and preferred responder (either a trusted community member trained in naloxone administration or Emergency Medical Services). Callers are then asked to ensure that they have their lights on, pets are put away and that their doors are unlocked to ensure a timely response. Once an emergency response plan is co-created, clients would use their substances and emergency responses would be enacted should the individual become unresponsive. A detailed call script can be found in Appendix 1. As the service progressed, it was found that the service provided many additional supports to clients beyond overdose prevention, including peer and mental health support and methamphetamine-induced psychosis de-escalation [31].

### Pre-implementation and pre-launch concerns

#### Legal review

A legal and ethical review was conducted in Alberta to determine the implications of NORS. Much of the review focused on determining how Canada’s Good Samaritan Act would function with such a service and the protections it would provide to substance users and service operators. The Good Samaritan Act exempts those who help someone in distress from legal action [32]. Grenfell Ministries independently received a legal review that suggested the need for liability and director’s insurance to protect the organization and staff members.

#### Stakeholder engagement

Given the large number of various stakeholders who engage with NORS, an appropriate stakeholder engagement strategy needed to be conducted. This included first responders, dispatch services, harm reduction advocates and agencies, public health officials, and most importantly people with lived and living experience of substance use. This not only helped inform how the line functioned, but also how to best implement the service in various jurisdictions and provinces. This was especially important for rural communities as it highlighted the concerns around EMS dispatch times, allowing us to utilize community-based call-outs as a means to navigate around this.

#### Establishing dispatch services

A major concern identified before launching the service was around the feasibility of dispatching EMS services across the country. Through initial conversations with dispatch services, it was determined that 90% of Canada had an interconnected dispatch system meaning someone could call from Vancouver British Columbia, and successfully have EMS services dispatched to the opposite side of the country such as Halifax, Nova Scotia. Despite this, there were still some parts of the country not covered by the interconnected dispatch service. Through discussion and investigation, it was determined that a service known as Northern 911, a dispatch agency that provides linkage to all dispatch centers in Canada (covering that additional 10%), was an effective service that could provide this reliability. They were able to support the launch of the service and became integral to its operation.

### Implementation of the service

#### Staffing

Hiring people with lived or living experience of substance use or those who have previously worked in the harm reduction sector was a key priority for the NORS line. Successful candidates received training via online modules around harm reduction, the structure of the organization, safer injection practices, overdose risk factors, and various specifics of service line operation. They also engage in virtual orientation sessions, practice calls, and buddy shifts. Currently, most new hires are former students or volunteers.

As the line operates 24/7 it was important to have 2–3 operators on the line at any time. Most staff work part-time (alongside several casual operators) as many had other jobs and responsibilities. If the line was busier than normal, the virtual staff room would be used to recruit volunteers who may be available to help field calls and provide support when needed. A short roster of approximately 5 volunteers is active to support the line. Operator shifts typically last 4–8 hours, depending on the availability of the staff and their personal preferences for hours. When additional support is required, operators can utilize the virtual staff room (a chat room) to request a client transfer, obtain resources for a caller, or seek the support of a supervisor or other colleagues. Debriefing was conducted with all operators following any adverse event such as an overdose/drug poisoning event, mental health crisis, or emotionally challenging situation.

Continued learning opportunities and group activities were provided to further a team atmosphere amongst the operators. These included sessions around support for survivors of incarceration, whether correctional or psychiatric, allowing individual operators to process any pain or trauma in a safe mutually supportive space. Other sessions involved focusing on dismantling anger, peer support circles, and establishing essential skills for operators to develop self-care and boundaries with clients. As the line matured, further sessions around diversity and inclusion were added based on requests from the peer operators. Later these virtual sessions opened up to clients and community members. Of note, the vast majority of these programs were provided by inkind support from Grenfell Ministries from another grant which was not renewed, limiting the capacity for this work to continue.

#### Software and data collection systems

In order to support the operators, a virtual staff room was established in which operators could communicate with each other about issues regarding client care and also provide mutual support for each other. A mobile phone app called “BAND” [33] was utilized for this as it has the ability to store essential reference documents that could be accessed by volunteers on their phones as needed. Noting the sensitive nature of the data collected during calls, all operators are instructed never to use client names and only refer to individuals by caller codes described in detail in Appendix 1.

Lastly, as operators work across the country in their own homes and using their own phones,  software known as Talkroute was leveraged which allows for call distribution. This Talkroute software allows for call routing, forwarding, and prioritizing along with text-based functions for both clients and operators, and is an essential operating system for our phone line.

### Policy and procedural implementation

While many lessons were applied from the previous Ontario Based OPH, the national scope of NORS required a revision of many of the established policies and procedures. Standard operating procedures were drafted along with documents outlining operational roles and responsibilities for operators and leadership. The manual was collaboratively codesigned with PWLLE and service operators and provided information with video and descriptive components.

While the initial focus of the NORS policies was around supportive virtual supervised consumption, they later focused on specific issues faced on the service line. For example, at times, individuals accessing the line did so for social services that put a strain on limited resources. As a result, operational policies were enacted limiting clients to four forty minute “peer support” type calls per day. Other processes were created around managing prank calls as well as lewd calls that occurred on occasion, as well as calls that were more aligned with poison control centers for accidental medication overdose. Additionally, due to the bilingual nature of Canada, an integrated French team with parallel policies and procedures was also created and implemented to support Francophone individuals.

Mental health psychosis and suicidal management were unexpected aspects of the phone line. To navigate this, specific training was created to help successfully de-escalate episodes of acute psychosis via the phone line to ensure the safety and well-being of the caller. These policies and procedures continue to be updated as an ongoing process.

### Funding and budget

While initially being run as a volunteer-based line, funding was eventually secured through Health Canada’s Substance Use and Addiction Programming. The funding was limited to piloting the NORS project, and establishing its initial feasibility as a national hotline to reduce overdose deaths. The funding was limited for a two-year period (April 2021–April 2023). Two-thirds of the budget was allocated towards human resources, including peer staff to operate the line 24/7. A portion of the funds was reserved for stakeholder feedback, training, capital costs around office supplies, computers, as well as research funding and marketing. Additional capital costs included shipping costs for marketing materials, line software costs, insurance, travel, and legal fees. There was funding allocated towards staff training as well as internal auditing expenses. These costs have been further described in a previously published cost-benefit analysis of the program [23]. Two separate grants from Health Care Excellence Canada and the Canadian Institute of Health Research provided additional research and evaluation support. There were additional reporting requirements to our funding agencies every 4 months. Funding for the French-speaking component of NORS was provided through Wellness Together Canada.

It should be noted that Grenfell Ministries was also leveraging funding from a separate program for in-kind support around the provision of learning opportunities and additional peer programming for employees, community members, and clients alike. Additionally, this other program provided capital costs for equipment like cell phones.

## Post-implementation services

### Marketing and outreach

**Fig. 1:**
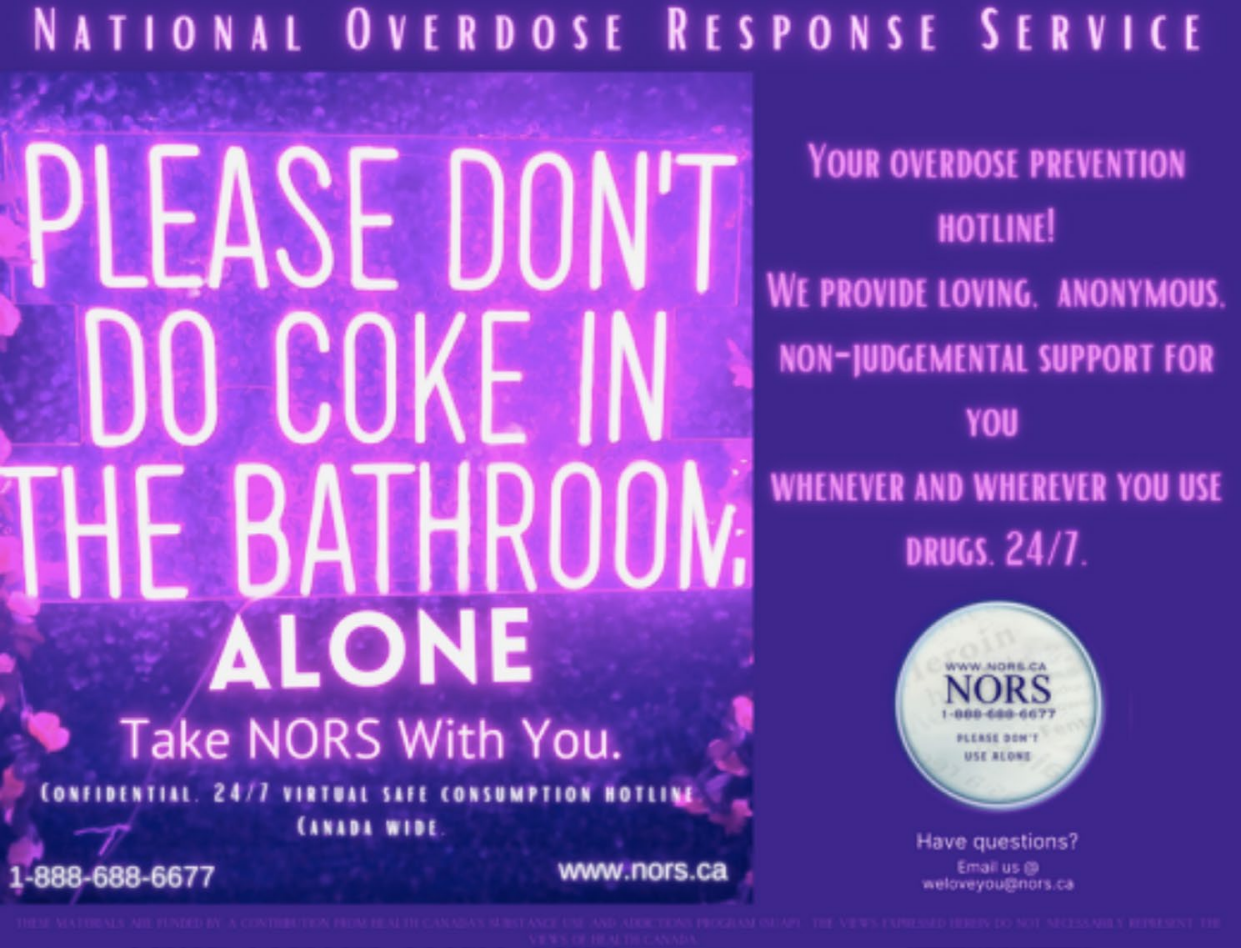
An example of NORS promotional material

An aggressive media campaign was conducted with NORS since inception. First, social media campaigns were utilized through paid advertisements, social media posts, and engagement activities on Facebook, Instagram, and Tiktok. The second involved advertisements on radio and TV. Promotional materials and merchandise were distributed to various agencies across Canada including information cards (Fig. 1), lighters, tumblers, and t-shirts. NORS staff members also hosted various Naloxone training sessions, with the ultimate goal of empowering communities to be their own response force.

### Social support sessions and group chat

After the launch of NORS, there were numerous requests from clients on the line, as well as the operators, for greater connections between clients and operators. This was particularly important during the peak of the pandemic and lockdown. In response to this, NORS created group-based activities facilitated by a moderator focused on promoting personal wellness for both substance users and those in recovery. It includes activities as well as sessions such as virtual yoga, meditation, breathwork, Qigong, tapping, and other somatic activities focused on strengthening mind/body connection reducing stress, and improving mindfulness. Additional activities, including trivia and games nights were also conducted to help form connections between users of the line as well as between the operators who work across the country. A group chat was also created to allow clients to communicate with each other.

### Research and evaluation

An evaluation and research team was created to ensure outcomes around the service were evaluated, areas for improvement were identified, and action was taken to improve the quality of service delivery. This was crucial to establishing the service’s scientific integrity and supporting efforts to obtain continued and possibly sustainable funding for the future.

## Results

### Service outcomes

Between December 15, 2020, and March 31, 2023, NORS operators recorded 6528 service calls, the majority of which (3994, 61.1%) were for virtual supervised consumption services followed by mental health and peer support (1703, 26.0%), and the remainder for operational support, and service inquiries [21, 22]. In another study of service use outcomes, of the 331 unique NORS clients, 57.7% lacked access to a physical supervised consumption site and 29.9% were unable to access the site due to either limited operational hours or lack of harm reduction support for their route of choice (most often smoking). Overall there were 77 drug poisoning events on the phone line which included either activation of emergency medical services or a community-based naloxone response, and lastly there were 3 false alarms where EMS was dispatched but there was no actual overdose event [21, 22]. Most significantly, despite the number of adverse events, there have been no deaths or prolonged hospitalizations with the line as confirmed by follow-up wellness checks conducted by NORS staff after an overdose event. Service users presented a variety of demographic backgrounds when accessing the service which are described in a recently published manuscript, highlights of which are provided below.

#### Social support sessions

From December 15th, 2020 to March 31, 2023, over 237 unique individuals attended various social support sessions/group wellness sessions provided by NORS.

### Marketing and outreach

The NORS Facebook page was heavily promoted, reaching over 91,808 individuals. The NORS Facebook page itself had garnered 1,520 followers with an additional 1,382 people who had liked the NORS page. Of these, 69% of them had identified as women and 28% identified as men. Women between the ages of 25 and 34 were the page’s most frequent visitors. Of the various visitors to the NORS site, the highest number of visitors came from Ontario, Alberta, British Columbia, and Quebec. NORS was informally promoted on Instagram gaining 1,061 followers, with 82% identifying as women, and 18% as men. TikTok gained over 711 followers within a three-month span of being launched. Between December 1st, 2022, to March 31, 2023, the NORS Google business profile has had 201 interactions, 1,123 views, and 1,437 searches, and maintained a 5-star rating. Much of the advertising also consisted of merchandise distribution. NORS campaigned by distributing shirts, lighters, stickers, and sweaters. Over 950 T-shirts, 240 coffee tumblers, 14,000 business cards, and 89 merch orders have been sent out across Canada. Over the course of 2.5 years, over 237 learning opportunities and sessions were provided to the NORS staff. The sessions were eventually opened up to community members and other organizations as well to participate in as part of an effort to further integrate NORS with the broader community to which it is a part.

### Funding

Since its inception, NORS has been supported by $1,922,759 in funding over two years. It was renewed for a third year for an additional $955,080.

## Discussion

Over the last two years, from conception to the implementation of the service, numerous lessons were learned. These lessons are grouped into categories including scope of service, organizational lessons, software and technology lessons, concerns with stakeholders and staffing, obtaining of funding, and metrics needed for evaluation and lastly improving exposure. One central theme around what we learned was that we needed to adapt to the needs of the individuals we served, including expanding the scope of our programming. This involved additional training to our staff and pivoting our policies and procedures. While this did put additional strain on our organization, it was central to our organization’s overall mission.

### Lessons learned during the implementation

#### Scope of service

While the line was initially created for virtual supervised consumption support, it became apparent that the line was being used for other purposes including peer based support, psychosis de-escalation, and education [31]. With psychosis de-escalation and other crisis events, staff group discussions helped create protocols around these complex areas. Where possible, NORS utilized case management support from its other programming provided for by a separate grant. These aspects were not initially considered when our staffing models were constructed and often went beyond the program’s scope. Members of the organization and funders had raised concerns that we were going beyond our original intentions. After a group discussion with our peer operating team, it was decided that we have to prioritize case managing these individuals as many of these services we were providing are often seen with other harm reduction services such as physical supervised consumption sites. It was also noted that many of our callers are high-frequency callers who are banned from all other crisis lines due to either policies surrounding substance use on crisis lines, callers calling other lines too frequently, or sexual arousal coinciding with substance use. It was felt that as these callers had nowhere else to turn to, so we as a line could not turn them away. To help manage these individuals’ boundary setting and call transfers were established should operators feel unsafe to manage these concerns while supporting access to low barrier harm reduction services.

One particular area of support that we did not anticipate was individuals who become highly stimulated and aroused from substance use. These individuals often become sexually explicit on the phone line. Not all of our operators and volunteers are comfortable with this aspect of the line, and boundaries need to be established and enforced as many peer operators have experienced sexual trauma and can be triggered. As such we have had to make conscious staff choices when these types of calls are started, sometimes transferring the caller to a staff member who is more comfortable with these types of interactions.  Lastly, based on more recent feedback, the line is starting to explore texting and webchat options to support callers.

### Organizational challenges and leadership

The scoping challenges which arose through operations, often conflicted with the variety of visions from the core founders. This led to several conflicts within the group. Prominent examples of this include: (1) Interpersonal issues (personalities) (2) Conflicts around the direction and goals of the service. (3) The Direction of Grenfell Ministries and how its work outside of NORS intersects with NORS. (4) A conflict of values and worldview due to team members coming from a variety of professional and living experience backgrounds with different approaches to problems (5) and lastly text-based communication leading to misunderstandings. To address this open and honest conversations were conducted amongst the leadership with a focus on consensus building to help ensure the program continued. Despite these issues, the service continued to thrive, and discussing these issues openly and candidly helped solidify the organization further. Weekly check-ins between leadership have also helped facilitate continued avoidance of conflict. While these were overall challenging, the experience has helped the leadership team grow even closer.

#### Software and technology

The BAND service app has been integral to the program’s operation, providing a sense of community between clients and operators. Features of this service allow for a private messaging channel that supports the operators. A portion of BAND also acts as a public-facing *community board.*

Several times during the implementation of the service, the telecommunication software of choice was pivoted. NORS currently utilizes *Talkroute*, which limits the number of operators who can use the software at a time. Additional concerns with Talkroute include its inability to queue calls or hold calls as needed. As the service expands, other technology platforms will need to be examined and incorporated. At this time we are exploring new options for operating services to meet the growing needs of the NORS program.

#### Staff training, communication, and volunteer retention

After exploring diverse educational strategies, dedicated staff teaching sessions were found to be optimal for learning and learner confidence. Prospective staff are encouraged to practice calls and study various service use outcomes to better prepare for scenarios and experiences that may arise on the line.

Communication between staff became a key component to the success of NORS. Given that the line is virtual, a virtual staff room/ chat group was created for staff to communicate with each other and line supervisors, as well as to further build a sense of community and to support staff wellness given the seriousness of the work around drug overdoses. Reflections from our staff have reinforced the need for staff to be in continuous engagement with each other, and it has helped NORS go beyond just an organization to being more of a family.

Volunteer retention continues to remain a challenge. Current audits and internal surveys cited “the busyness of life” as being the main reason for their attrition.

#### Referrals

A key issue noted by both clients and staff was that not all external referrals were successful. Many callers tried using suggested referral sources but were not always satisfied with these services as some lacked trauma-informed approaches. As such, efforts were made to appropriately vet resources by NORS staff and keep an ongoing catalog of tried and tested services that were in line with NORS values. These resources continue to be tested and vetted with clients and staff to support our callers [34].

### Post implementation lessons

#### Data metrics and evaluation

Crucial to the establishment of NORS was the need to demonstrate the effectiveness of the service to funders as well as the broader public health, public policy, and scientific communities. Given the sensitivities around the data from the substance-using community, internal discussions were conducted to decide what data was acceptable and not acceptable to collect to ensure privacy was not compromised. Additionally, a consensus building exercise was conducted with others in the sector to determine the best metrics to evaluate [35]. Discussions around data privacy are necessary for the establishment of similar services and are integral to ensuring trust between the service and the users [36].

#### Creating exposure around the service

Initial attempts to advertise NORS included TV advertisements and radio ads, social media advertising and engagement along with merchandise distribution. NORS’ greatest success however came from the attendance at various public events, training around naloxone kit usage, and messaging on not using alone [37]. Presentations and conferences were used within the scientific community to create further exposure and understanding of the service. Lastly, NORS staff conducted a national outreach program to discuss NORS with various organizations across the country. These organizations were found through Google searches, cold calls, and snowball connections between agencies. Lastly, speaker engagement sessions, were frequently leveraged to educate front-line workers on the program.

#### Funding challenges

Funding continues to be a large challenge with the service. The initial funding was to test the feasibility of the program as a pilot. While additional funding has been provided to extend the project by a year, additional funds are needed around other aspects of the program beyond just supervised consumption. For instance, as previously mentioned, much of the staffing education and wellness support programs were funded through another grant supporting the NORS parent company Grenfell. In addition to this, the other grant also provided case management support to NORS clients where needed. To date, no permanent funding has been provided for this service.

## Conclusion

The start of North America’s first national overdose prevention service was met with many unique lessons and opportunities. We hope these lessons can be used to support others in the implementation of this service in their own jurisdiction and provide support for individuals using substances alone.

## Data Availability

The datasets generated and/or analysed during the current study are not publicly available due privacy concerns around NORS clients’ illicit substance use but are available from the corresponding author on reasonable request.

## Appendix 1

### Part 1

Hello, Overdose Response Service, this is ___ speaking.

Are you a new or returning caller?

### Returning caller

As you know I can’t access your location so I will take your word for it and let us walk through the information I will collect quickly.

- What is your caller code?
- Your address?
- Is this an apartment? If so what is the code to get into the building?
- Where are you located in the building?
- What type of drug are you using?
- How will you be using that today?
- Who would you like us to call in case of an emergency?
- Is there a phone number you would like us to call if we are disconnected for any reason?

Just a few reminders I would like to mention. Make sure that you have your lights on, naloxone is available, and all your drugs and paraphernalia are put away, please ensure any pets are locked away in case anyone needs to enter your home as well.

Alright, that should be everything! Go ahead and I am happy to either chat with you or just check in periodically to ensure that you are still safe.

### New caller

We are not able to directly access your location so I will be taking your word for it. I just want to ask you a few questions to help in the case of emergency response and for research data collection and this will not link you to our services personally.

This service is completely confidential except for a few different cases like harm to yourself or someone else. Is that OK with you?

Can I ask where you heard about us?

We usually will use a caller code to know who we are chatting with which comprises the first two letters of your first name and last name as well as your year of birth. For example, my name is Will and my caller code is WIRI00. You can feel free to make up any nickname you want as long as you remember it.

- What is your caller code?
- Your address? - Again as a reminder, we will only use this information if there is an emergency and it is not recorded anywhere.
- Is this an apartment? If so what is the code to get into the building?
- Where are you located in the building?
- What type of drug are you using?
- How will you be using that today?
- Who would you like us to call in case of an emergency?
- Is there a phone number you would like us to call if we are disconnected for any reason?
- Alright, that should be everything! Go ahead and I am happy to either chat with you or just check in periodically to ensure that you are still safe.
